# Very high commutation quality factor and dielectric tunability in nanocomposite SrTiO_3_ thin films with *T*_c_ enhanced to >300 °C†
†Electronic supplementary information (ESI) available. See DOI: 10.1039/c7nr06991j


**DOI:** 10.1039/c7nr06991j

**Published:** 2018-02-05

**Authors:** Abhijeet L. Sangle, Oon Jew Lee, Ahmed Kursumovic, Wenrui Zhang, Aiping Chen, Haiyan Wang, Judith L. MacManus-Driscoll

**Affiliations:** a Department of Materials Science and Metallurgy , University of Cambridge , UK . Email: sangle.abhijeet@gmail.com ; Email: jld35@cam.ac.uk; b School of Fundamental Science , Universiti Malaysia Terengganu , 21300 Kuala Terengganu , Malaysia; c Center for Functional Nanomaterials , Brookhaven National Laboratory , Bldg. 735 – P.O. Box 5000 , Upton , NY 11973-5000 , USA; d Center for Integrated Nanotechnologies (CINT) , Los Alamos National Laboratory , Los Alamos , NM 87545 , USA; e School of Materials Engineering , Purdue University , West Lafayette , IN 47907 , USA

## Abstract

We report on nanoengineered SrTiO_3_–Sm_2_O_3_ nanocomposite thin films with the highest reported values of commutation quality factor (CQF or *K*-factor) of >2800 in SrTiO_3_ at room temperature. The films also had a large tunability of dielectric constant (49%), low tangent loss (tan *δ* = 0.01) and a Curie temperature for SrTiO_3_ > 300 °C, making them very attractive for tunable RF applications. The enhanced properties originate from the unique nanostructure in the films, with <20 nm diameter strain-controlling Sm_2_O_3_ nanocolumns embedded in a SrTiO_3_ matrix. Very large *out-of-plane* strains (up to 2.6%) and high tetragonality (*c*/*a*) (up to 1.013) were induced in the SrTiO_3_. The *K*-factor was further enhanced by adding 1 at% Sc^3+^ (acceptor) dopant in SrTiO_3_ to a value of 3300 with the tangent loss being ≤0.01 up to 1000 kV cm^–1^.

## Abstract 2

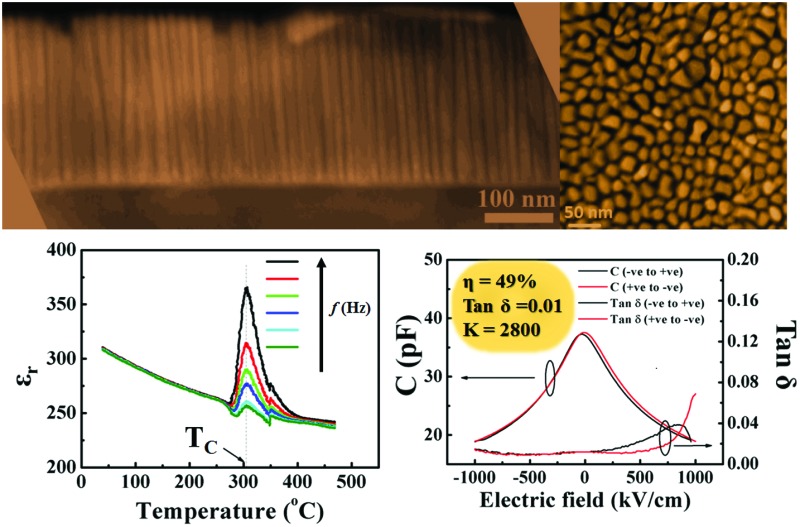
We report on nanoengineered SrTiO_3_–Sm_2_O_3_ nanocomposite thin films with the highest reported values of commutation quality factor (CQF or *K*-factor) of >2800 in SrTiO_3_ at room temperature.

## 1. Introduction

Ever since the demonstration of radio signals for communication by Marconi in 1901,^1^ there has been a tectonic shift in communications technologies. At the heart of the modern day mobile communications technologies are tunable microwave devices such as filters, phase shifters, delay lines, varactors, resonators, variable power dividers and frequency oscillators.^2^–^4^ The performance of tunable microwave devices is measured using several different yardsticks such as dielectric tunability (*η*) (defined as the percentage change in dielectric permittivity upon application of a dc electric field compared to the dielectric permittivity at no external dc electric field), dissipation loss (quantified by the loss tangent, tan *δ*) and a net *Q* factor (1/tan *δ*). The total dissipation loss can be due to contribution from different factors such as pure dielectric loss, series resistances of connecting wires, *etc*. While a high dielectric tunability coupled with low tangent loss is normally required, Vendik *et al.*^5^ have provided a useful commutation quality factor (CQF) or *K*-factor which incorporates tunability and dielectric loss in a single relation as follows:1
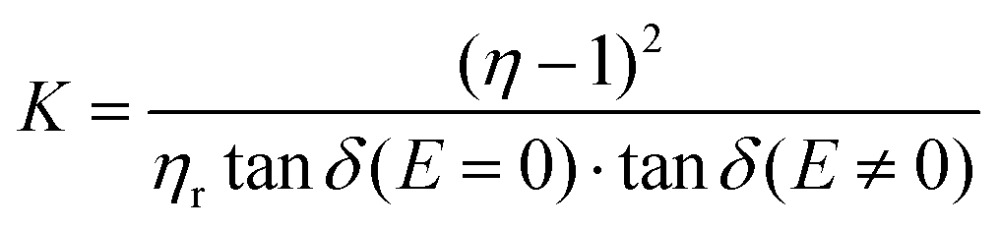
where, *η*_r_ is the ratio of dielectric permittivity at a non-zero dc electric field to that at zero dc electric field. Tagantsev *et al.* have stated a requirement of CQF > 900 to satisfy the need of contemporary tunable microwave devices.^6^

Ferroelectrics can offer an excellent alternative to the conventional semiconductor-based tunable microwave devices.^2^,^6^ They show many potential benefits such as high tunability, low loss, high tuning speed, small leakage currents, low power consumption, radiation hardness and high breakdown electric field, making them very attractive for use in communications.^2^,^7^,^8^ Over the last five decades or so, ferroelectrics have been studied in different forms such as pure or doped,^6^,^9^ composites or single-phase systems^10^ and bulk or films.^11^ Sometimes, these different forms have been combined, *e.g.*, doped ferroelectric containing composite films.^12^ Out of these different forms, bulk materials face major drawbacks such as the need of high tuning voltages and large sizes, which are not ideal for the increasingly smaller communication devices.^11^ These disadvantages can be removed by growing ferroelectric thin films, wherein the required high electric field is generated at a fraction of the voltage needed for the bulk devices. Their small size also gives the possibility of integrating them into conventional microwave technology.^11^ Ferroelectric perovskites such as PbZr_*x*_Ti_1–*x*_O_3_, BaTiO_3_ and Ba_*x*_Sr_1–*x*_TiO_3_ (BSTO) are widely used for tunable microwave devices. However, they face several challenges. Lead-based ferroelectrics such as PbZr_*x*_Ti_1–*x*_O_3_, PbTiO_3_, Pb_*x*_La_1–*x*_Zr_*y*_Ti_1–*y*_O_3_ are increasingly discouraged by the environmental agencies owing to the toxicity of Pb.^13^,^14^ Ba_*x*_Sr_1–*x*_TiO_3_ (BSTO) is beneficial over BTO because it has a relatively low loss while maintaining a high tunability,^15^ but it does not exhibit both these optimised properties at the same time. Indeed, BSTO has a broad ferroelectric-paraelectric phase transition over hundreds of degrees Celsius, deteriorating its tunable microwave performance.^16^ It has been suggested that the heterogeneity associated with the chemical substitution of Sr^2+^ in BaTiO_3_ lattice is responsible.^16^ Moreover, BSTO usually possesses a very high dielectric constant.^17^,^18^ This is not suitable for impedance matching,^6^,^19^–^21^ high power phase shifters, or high power accelerating structures with dielectric loading,^22^ where low dielectric permittivity is needed *in addition* to high tunability and low loss. It has been estimated that the dielectric constant (relative permittivity (*ε*_r_)) should be <500 for impedance matching purposes.^20^,^21^

On the other hand, the incipient ferroelectric SrTiO_3_ (STO) can offer a viable solution to the problem. STO has low permittivity (*ε*_r_ = 300 for bulk STO at room temperature^23^). However, owing to its bulk ferroelectric *T*_c_ of <100 K, STO is useful only for low temperature applications.^24^,^25^ At room temperature, STO has low tunability^24^ because here it is a cubic non-ferroelectric, rather than a highly tetragonal ferroelectric which is needed to obtain high tunability.^16^,^24^,^26^ If high tetragonality can be induced in STO at room temperature its ferroelectric properties can be maintained. Indeed, Haeni *et al.* achieved room temperature ferroelectricity in STO by epitaxial straining of 70–80 nm thin film by clamping it to a substrate.^16^ They achieved a very high tunability of 70% at room temperature, and at 10 GHz.^27^ However, the loss was high at 0.067, which is relatively high, yielding an overall CQF of ∼360.

The aim of this work is to produce a superior microwave tunable material based on STO. With the very strong benefit of much lower dielectric constant than analogue perovskite ferroelectrics, SrTiO_3_ has the potential to be an ideal microwave tunable material. Two challenges need to be overcome. First, since in ferroelectric materials the highest tunability is close to the Curie temperature, for operation at room temperature or higher, *T*_c_ needs to be enhanced to well above room temperature.^16^,^28^,^29^ Second, the loss must be kept low. An enhanced *T*_c_ in STO films of practical thickness has never been shown before and neither has reduced loss been shown in such films.

The novelty of the work lies in the engineering of STO thin films using a combined nanocomposite and doping approach, to give properties which cannot be achieved by any other method. As we show later, our films have the highest values of commutation quality factor ever reported at room temperature.

We use nanocomposite films to achieve high vertical tensile strain in the films independent of the substrate. Using the nanocomposite approach, there is no intrinsic limitation on strain control of thickness (with micron thick films possible). This is in strong contrast to standard, plain epitaxial films where strain is controlled by the substrate, at least for the first few 10's of nm, with gradual relaxation occurring above this up to ∼100–200 nm. As we have shown before for BTO/Sm_2_O_3_ films^31^ and BSTO/Sm_2_O_3_ films,^26^ the nanocomposite approach allows us to maintain ferroelectricity to a temperature well above room temperature in micron thick films. Strain is controlled in a direction perpendicular to the substrate using stiff Sm_2_O_3_ nanocolumns which grow by self-assembly in the STO matrix, with Sm_2_O_3_ substituting only minimally in the titanate ferroelectrics.^30^ Sm_2_O_3_ is a low-loss, low permittivity, passive dielectric material which does not show any tunability with electric field. The tunability in the STO is expected to be controlled solely by the strain induced in it by the presence of the stiff Sm_2_O_3_.^26^

Achieving low loss in STO could be very challenging, because of possible formation of oxygen vacancies while straining, and yet to achieve a high *K*-factor, it is very important to pay attention both to increasing *η* and lowering loss. To address the potential loss problem from oxygen deficiency, we explored acceptor-doping of the lower valent ion (Sc^3+^) onto the Ti^4+^ site. Leakage has been shown to be lower in such SrTiO_3_ and Ba_*x*_Sr_(1–*x*)_TiO_3_ films than in standard plain films.^9^,^32^

## 2. Results and discussion

**Fig. 1:**
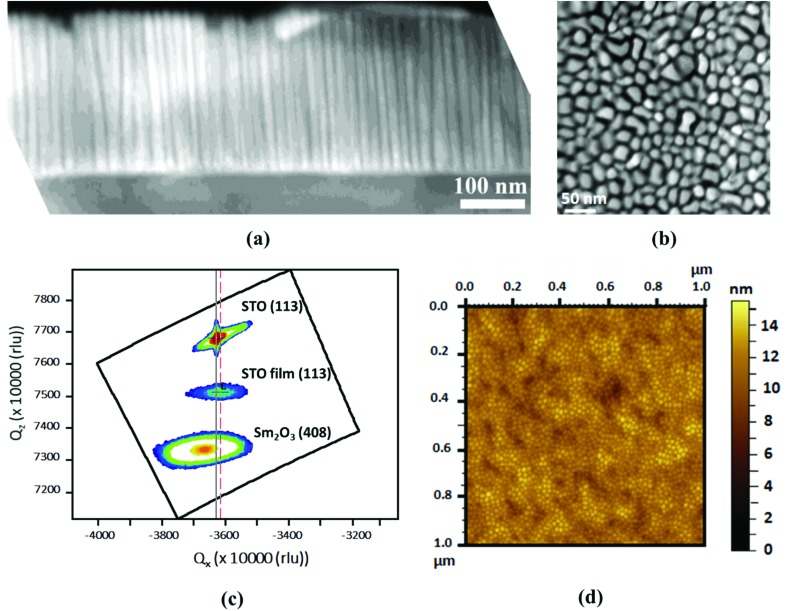
(a) Cross-section and (b) plan-view STEM of a ∼300 nm thick SrTiO_3_–Sm_2_O_3_ (30–70 wt%) film grown on SrRuO_3_/SrTiO_3_, showing vertical Sm_2_O_3_ nanocolumns embedded in SrTiO_3_ (STO) film matrix. (c) Reciprocal space map around the STO (113) substrate peak showing that the STO film is strained *out-of-plane* as evidenced by the lower *Q*_*z*_ value of the (113) STO film peak, (d) AFM image showing smooth film surface.

Fig. 1a and b show cross-sectional and plan-view of scanning transmission electron microscope (STEM) images, respectively, of a nanocomposite film with 70 wt% Sm_2_O_3_. The STEM images were taken under the high angle annular dark field mode where the image contrast is proportional to *Z*^2^. It is clear that the films contain vertical Sm_2_O_3_ nanocolumns of diameter ∼15–16 nm (with brighter contrast) interspersed in a STO matrix (in darker contrast).

The phases in the film are both aligned along (001), with STO being aligned *in-plane*, cube-on-cube, and the Sm_2_O_3_ being rotated by 45° *in-plane*, both as expected.^26^,^31^ The X-ray information on the films is shown in Fig. S1a and S1b in the ESI.†

Fig. 1c shows an X-ray reciprocal space map around the STO (113) peak. Here, the SrTiO_3_ film peak is clearly separated from and lower than the STO substrate peak along *Q*_*z*_ (*i.e.*, in the *out-of-plane* direction). On the other hand, the film peak is nearly fully *in-plane* strained to the substrate, as seen by the very close overlap of the *Q*_*x*_ peak positions. Thus, the strong vertical strain-controlling effect of the stiff Sm_2_O_3_ scaffold nanopillars on the SrTiO_3_ film matrix is evident. The nanocolumns of Sm_2_O_3_ dispersed in the SrTiO_3_ matrix can be identified from the atomic force microscope (AFM) image shown in Fig. 1d. The films are very smooth, the root-mean-square (RMS) roughness being around only 4.5 nm.

**Fig. 2:**
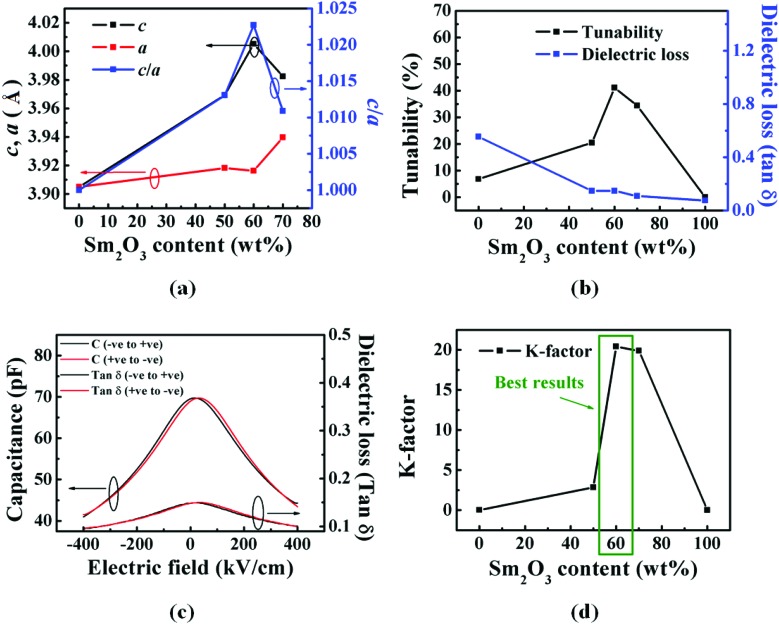
(a) The variation of *out-of-plane* (*c*) and *in-plane* (*a*) lattice parameters of SrTiO_3_ as well as tetragonality (*c*/*a* ratio); (b)–(d) shows dielectric properties of ∼250 nm thick films grown on SrRuO_3_/SrTiO_3_. (b) Dependence of tunability at 400 kV cm^–1^ and loss tangent (at 0 kV cm^–1^) on Sm_2_O_3_ content in the films; (c) relative permittivity (*ε*_r_) and tan *δ vs.* Electric field data for films of 60 wt% Sm_2_O_3_; (d) dependence of commutation quality factor (*K*-factor) for an electric field of 500 kV cm^–1^ on Sm_2_O_3_ content in the films. [*Note*: The tunability, tangent loss and *K*-factor for Sm_2_O_3_ wt% of 0 and 100 were calculated for 80 kV cm^–1^ electric field].

The structural and dielectric properties of the films with varying Sm_2_O_3_ content (from 0 to 100 wt%) in them are shown in Fig. 2.

The STO *out-of-plane* lattice parameter (*c*) increased from 3.905 Å for pure STO up to a maximum of 4.005 Å for 60 wt% Sm_2_O_3_ films (Fig. 2a). This behaviour can be attributed to greater interfacial area with increasing Sm_2_O_3_ in the film, hence availing more fraction of material at the interface for vertical straining.^26^ For high volume fractions of Sm_2_O_3_, the nanopillars will begin to be connected and the interfacial area with the SrTiO_3_ will reduce, thus explaining the reduction in *out-of-plane* lattice parameter above 60 wt% Sm_2_O_3_. Similar results have been obtained before.^33^ The *in-plane* lattice parameter (*a*) also increased from the pure film to the 60 wt% Sm_2_O_3_ film, albeit to a much lesser extent, from 3.905 Å for pure SrTiO_3_ to 3.916 Å for the 60 wt% Sm_2_O_3_ film (Fig. 2a). The increase in *in-plane* lattice parameter can be attributed to the restricted thermal contraction, upon cooling of the SrTiO_3_ matrix (because it is epitaxially pinned by the stiff Sm_2_O_3_ nanopillars) post-growth, leading to ‘auxetic-like’ behaviour as modelled previously for the Ba_(1–*x*)_Sr_*x*_TiO_3_/Sm_2_O_3_ system.^34^ A maximum tetragonality of 1.023 was achieved here which is very high compared to 1.014 obtained by Haeni *et al.*^16^ by bi-axial *in-plane* straining of their films to substrates.

The tunability of the dielectric constant was computed from the capacitance *vs.* electric field curves. A small AC disturbance of 50 mV amplitude and electric field of 400 kV cm^–1^ were used. The dielectric tunability peaked with Sm_2_O_3_ content (Fig. 2b) coincident with the peak in tetragonality (Fig. 2a), underscoring the importance of tetragonality in determining the tunability of a ferroelectric material. The increased tunability with increasing tetragonality can be explained by the increased *c* lattice parameter. Hence, there is increased space along *c* (*out-of-plane* direction) for the vibration of Ti^4+^ ions in the oxygen tetrahedron of a SrTiO_3_ unit cell. The relative permittivity (*ε*_r_) is directly related to the electrical susceptibility (*χ*_e_ = *ε*_r_ – 1), *i.e.* it reflects the ability of the material to polarise upon application of an external electric field. This tendency of the material to be polarised (*i.e.* electrical susceptibility) is highest at zero external dc bias field, as there is plenty of room for Ti^4+^ to be pushed from its central position in the direction of external electric field. With increasing *c*/*a* ratio, the electrical susceptibility of the material, at zero external dc bias field, increases, because of increasingly larger room available for Ti^4+^ to be pushed in the direction of applied external electric field. Similar behaviour has been observed by Hyun and Char^35^ and showed computationally by Antons *et al.*^36^

The inverse correlation of tunability and loss is a very beneficial aspect of the composite films, since normally these two parameters directly correlate.^26^ However, for the *x* = 0.6 sample, the loss values are rather high at 0.15. Fig. 2c shows capacitance *vs.* electric field data for the optimum composition film (60 wt% Sm_2_O_3_). The presence of butterfly-shaped loops confirms the presence of ferroelectricity.^37^ As expected, the relative permittivity of the columnar nanocomposite films decreased with increasing Sm_2_O_3_ content due to the lower permittivity of Sm_2_O_3_ (Fig. S3 in ESI†).

The *K*-factor follows the same dependence on Sm_2_O_3_ fraction (Fig. 2d), similar to the tunability relation (Fig. 2b). However, for a 400 kV cm^–1^ applied field, the highest *K*-factor achieved was 20. The *K*-factor and tunability further increased to 53 and 57%, respectively, upon increasing the maximum dc electric field to ∼1000 kV cm^–1^. While the tunability value is one of the best for SrTiO_3_, and also comparable to literature reports for other ferroelectric thin films,^8^,^38^ the *K*-factor is insufficiently high for practical applications, where >900 is a target value.^6^ Therefore, subsequent experiments were focussed on reducing the loss (and hence on enhancing *K*).

It has been reported that the interface between the electrode and the film plays a crucial role in deciding the loss, with smooth, epitaxial and highly crystalline interfaces being necessary for minimising the loss.^9^ The presence of disorder at the interface, even at the smallest scale, can cause significant loss^39^ as atomistic defects at the interface scatter the electric field.^39^ Even though the SrRuO_3_ electrode was smooth (RMS roughness ∼0.30 nm) and highly conductive (280 μΩ cm), and the nanocomposite film grown on it was highly epitaxial, the SrRuO_3_ film is granular (grains and grain boundaries) which means there will be more defects at the interface with the composite film.

**Fig. 3:**
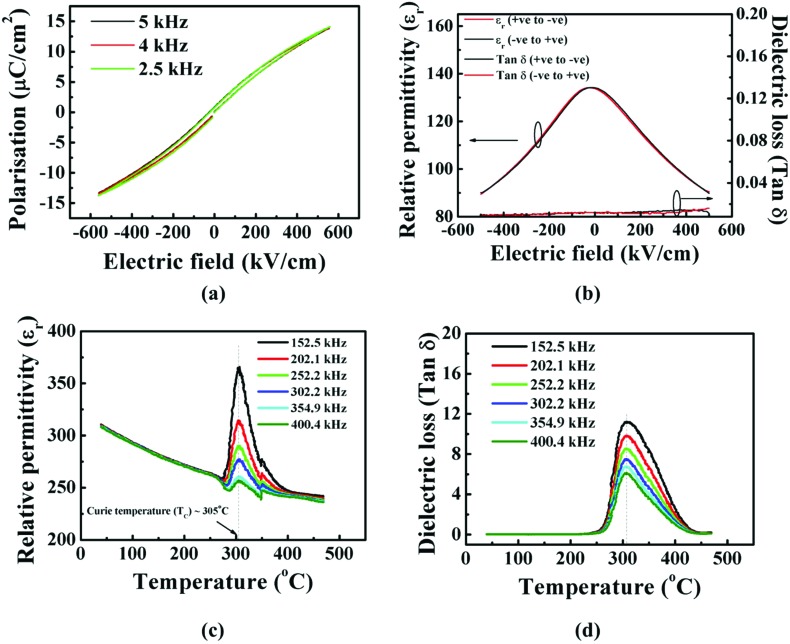
Ferroelectric properties of ∼250 nm STO films with 60 wt% Sm_2_O_3_ addition grown on Nb-STO substrates. (a) Room temperature polarisation (*P*) *vs.* electric field (*E*) hysteresis loops (PE loops) at different frequencies; (b) relative permittivity (*ε*_r_) and tan *δ vs.* electric field (*E*) behaviour; (c) temperature dependence of relative permittivity (*ε*_r_) and (d) temperature dependence of the dielectric loss (tan *δ*).

Hence, the nanocomposite films were grown on conducting (001) Nb-doped single crystal STO (Nb-STO) substrates which also served as bottom electrodes, while at the same time giving a more perfect homoepitaxial interface. Hence, a 250 nm thick film of the optimum 60 wt% Sm_2_O_3_ composition was grown on Nb-STO. Fig. 3a shows polarisation-electric field (PE) loops. The lack of frequency dispersion in the PE loops rules out a relaxor character of ferroelectricity.^40^ The narrow shape of the PE loop can be attributed to finer lateral dimensions leading to formation of smaller domains.^41^,^42^

The variation of capacitance with electric field for the films is shown in Fig. 3b. From these curves, the calculated tunability was 33% and the *K*-factor was 1500 (at 1 MHz, 500 kV cm^–1^ and at room temperature). The tunability and *K*-factor further increased to 49% and 2800, respectively, for 1000 kV cm^–1^ (see Fig. S2b in ESI†). To the best of the authors’ knowledge, this is the highest ever reported *K*-factor at 1 MHz and at room temperature for SrTiO_3_ in any form – pure or doped, single phase or composite and bulk or thin/thick film. We see from Fig. 3b (and Fig. S2b†) that the loss tangent is roughly an order of magnitude less than what we have observed with the SrRuO_3_ bottom electrode, indicating the strong influence of the quality of the nanocomposite/electrode interface.

The polarisation *vs.* electric field (PE) loops (shown in Fig. S2a in ESI†) showed the ferroelectric nature of the films to at least 270 °C, after which the loops became too lossy to conclude whether the films were ferroelectric or paraelectric. The variation in relative permittivity (*ε*_r_) and loss tangent (tan *δ*) (Fig. 3c and d, respectively) with temperature show peaks at 305 °C with no frequency dispersion of the peak positions with temperature, confirming no relaxor type ferroelectricity in these films.^40^ The results corroborate the high ferroelectric temperature determined from the PE loops. This is the highest ever reported Curie temperatures for SrTiO_3_.

Finally, to further reduce the loss in the ferroelectric films, Sc^3+^ doping of the SrTiO_3_ films was undertaken at the doping level of 1 at% on B-site. Sc^3+^ in place of Ti^4+^ is expected to produce acceptor doping to compensate electronic doping arising from the formation of oxygen vacancies. Indeed, Fe^2+^, Fe^3+^, Sc^3+^, Mg^2+^, Mn^3+^, *etc*. have previously been studied as dopants for loss reduction with varying degrees of success.^43^–^45^ The preparation of the Sc-doped SrTiO_3_ ceramic powder for the target is described in the methods section.

**Fig. 4:**
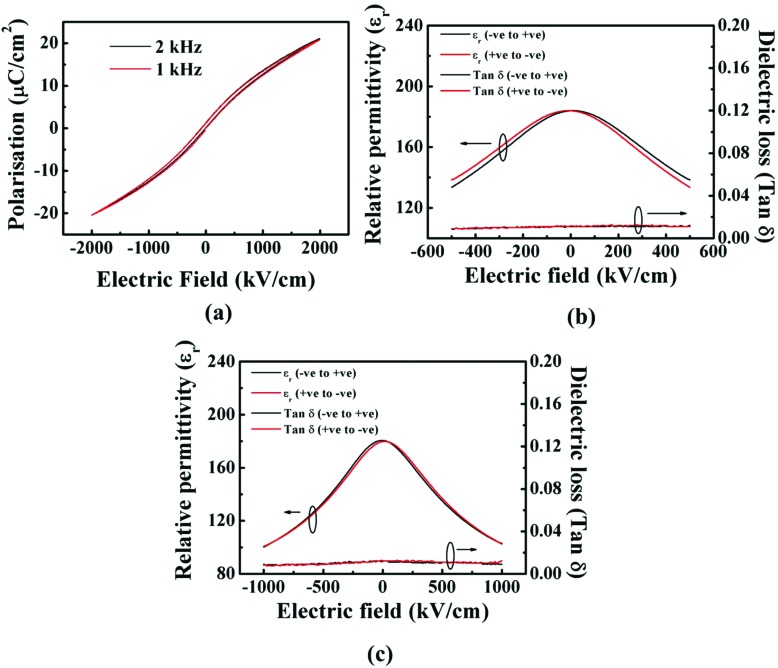
Electrical characterisation results of 250 nm thick, 1 mol% Sc^3+^ doped SrTiO_3_–Sm_2_O_3_ (60 wt% Sm_2_O_3_) film grown on Nb–SrTiO_3_ substrate. (a) Room temperature polarisation (*P*) *vs.* electric field (*E*) hysteresis measurements performed at different frequencies, (b) and (c) relative permittivity (*ε*_r_) and tan *δ vs.* electric field measurements at 500 kV cm^–1^ and 1000 kV cm^–1^, respectively.

As shown in Fig. 4a, the Sc-doped SrTiO_3_ films were found to be ferroelectric with identical PE loops obtained for different frequencies. A tunability of 45% was obtained at 1000 kV cm^–1^ electric field (as determined from capacitance *versus* electric field plots, Fig. 4b and c). A loss tangent of ≤0.01 (Fig. 4b and c) at all electric fields. This gives a *K*-factor of 3300. The tunability is similar as for the undoped STO nanocomposite films. The lower loss of ≤0.01 across the whole field range, compared to the undoped film led to an increase in the *K*-factor to 3300. Table 1 compares the high frequency properties of our columnar nanocomposite films measured at room temperature with some of the best results reported in the literature.

**Table 1:** Comparison of tunable microwave performance of the devices studied in this work with some of the best data from the literature (*η* is the tunability as defined before)

Device	Tunable high frequency performance (*η* in %)	Frequency	Ref.
SrTiO_3_–Sm_2_O_3_ columnar composite films	*η* = 49, tan *δ* ≤ 0.02	Measured at 1 MHz and room temperature	This work
	*K* = 2.8 × 10^3^, *ε*_r_ = 135		
(1 at%) Sc-doped SrTiO_3_–Sm_2_O_3_ columnar composite films	*η* = 45, tan *δ* ≤ 0.01	Measured at 1 MHz and room temperature	This work
	*K* = 3.3 × 10^3^, *ε*_r_ = 180		
SrTiO_3_ thin film, molecular beam epitaxy (MBE) grown	*η* = 70, tan *δ* = 0.067	Measured at 10 GHz and room temperature	27
	*K*(estimated) ∼ 3.6 × 10^2^, *ε*_r_ ∼ 3000		
Ba_(1–*x*)_Sr_*x*_TiO_3_ thick film	*η* = 60, tan *δ* = 0.01	Measured at 1 MHz and room temperature	46
	*K* ∼ 1.8 × 10^4^, *ε*_r_ ∼ 270		
Ba_(1–*x*)_Sr_*x*_TiO_3_ thin film, PLD grown	*η* = 45, tan *δ* = 0.0057	Measured at 1 MHz and room temperature	47
	*K* = 1.5 × 10^4^, *ε*_r_ = unknown		
Mn-doped Pb_(1–*x*)_Sr_*x*_TiO_3_ thin film, sol–gel grown	*η* = 70, tan *δ* = 0.03	Measured at 100 kHz at room temperature	48
	*K* = 2.1 × 10^3^, *ε*_r_ ∼ 1000		
Ba_(1–*x*)_Sr_*x*_TiO_3_–Bi_1.5_ZnNb_1.5_O_7_ (80–20) (probably) granular composite thin film, PLD grown	*η* > 90, tan *δ* = 0.007	Measured at 1 MHz and room temperature	49
	*K* = 1.16 × 10^6^, *ε*_r_ ∼ 200		
Ba_(1–*x*)_Sr_*x*_TiO_3_–Sm_2_O_3_ (25–75 wt%) columnar composite film, PLD grown	*η* = 75, tan *δ* < 0.01	Measured at 1 MHz and room temperature	26
	*K* = 8–9 × 10^5^, *ε*_r_ ∼ 4000		
Bi_1.5_ZnNb_1.5_O_7_ – Mn-doped Ba_(1–*x*)_Sr_*x*_TiO_3_ laminated composite film, PLD grown	*η* = 60, tan *δ* < 0.005	Measured at 100 kHz and room temperature	12
	*K* = 9.0 × 10^4^, *ε*_r_ ∼ 400		

The columnar STO nanocomposite films of this work have the highest reported *K*-factor for STO. While better *K*-factors have been reported for Ba_*x*_Sr_(1–*x*)_TiO_3_ (BSTO)-containing films (Table 2), the SrTiO_3_ composite films are more suited for applications which require lower dielectric permittivity *e.g.* high power phase shifters or high power accelerating structures with dielectric loading or impedance matching in complex circuits.

**Table 2:** Growth conditions explored for growth of heteroepitaxial (Sc-doped) SrTiO_3_–Sm_2_O_3_ nanocomposite films

Parameter	Value
Deposition temperature	750–800 °C
Oxygen gas pressure during the deposition	0.2 mbar
Laser fluence	∼2.05 mJ cm^–2^
Laser pulse frequency	1 Hz
Deposition time	25 minutes for (∼250 nm thick films)
Post-annealing conditions	650 °C, 400 mbar O_2_ partial pressure and annealing time of 60 minutes

Finally, we note that the relative permittivity, *ε*_r_, was around 30% higher for the Sc-doped samples. This 30% increase in *ε*_r_ is not significant when experimental error is taken into account (from run to run, film thickness, variation in Pt pad area, and difference in amount of remnant Ag paint on the substrate bottom, Ag being used to glue the substrate to the heater during growth).

## 3. Conclusion

In summary, we have grown heteroepitaxial nanocomposite thin films of SrTiO_3_–Sm_2_O_3_ on SrTiO_3_. Via vertical epitaxy of the SrTiO_3_ film using stiff, <20 nm-sized Sm_2_O_3_ nanopillars embedded in it, we have achieved very high *out-of-plane* strain values of 2.6%, enabled by the strong epitaxial vertical clamping *out-of-plane* and tetragonality values of 1.013. The high tetragonality meant that the ferroelectric Curie temperature was raised to >300 °C, the highest ever reported in SrTiO_3_. The dielectric tunability at room temperature was ∼49% at room temperature. The loss tangent was ≤0.01, thus giving a commutation quality factor of 2800. Acceptor doping by Sc^3+^ led to further improvement in commutation quality factor to 3300. This value is well above the recommended value of commutation quality factor (900), a value which has not been achieved previously in standard SrTiO_3_ thin films. Moreover, the dielectric constant is in the acceptable limit (*ε*_r_ < 500) for applications such as impedance matching for high power phase shifters, or for high power accelerating structures with dielectric loading, thus making the composite films attractive for tunable microwave devices.

## 4. Experimental section

Columnar nanocomposite films of SrTiO_3_ or Sc-doped SrTiO_3_, with Sm_2_O_3_ of different weight fractions from 0 to 70 wt% were prepared by pulsed laser deposition on SrRuO_3_-buffered SrTiO_3_ single crystal substrates (SrRuO_3_ acting as an electrode layer) or directly on Nb-doped SrTiO_3_. The SrRuO_3_ served as a conducting electrode layer and was grown from a commercial SrRuO_3_ target. The SrRuO_3_ films were grown at 700 °C, in an off-axis geometry in 0.2 mbar O_2_ atmosphere at 9.8 sccm flow of O_2_ gas. The films of ∼50 nm thickness were post-annealed at 450 °C for 1 hour and in 400 mbar O_2_ atmosphere. The columnar nanocomposite films were prepared under the conditions mentioned in Table 2. A range of conditions explored in order to optimise tunability and commutation quality factor (*K*) by maximising the *out-of-plane* strain while keeping the RMS roughness low.

The targets used for the SrTiO_3_ nanocomposite films were made by standard ceramic procedures. Pure SrTiO_3_ and Sm_2_O_3_ powders were mixed in the desired proportion and finely ground to create a homogenous mixture. This powder mixture was then uniaxially pressed under a >100 kN force and then sintered at ∼1100 °C for 6 hours. Sc-doped SrTiO_3_ powder was prepared by mixing SrCO_3_, TiO_2_ and Sc_2_O_3_ powders in the desired stoichiometric proportion followed by grinding and pressing, as before. These pellets were then calcined at ∼900 °C for ∼6 hours and reground again into fine powders and pressed and calcined again. The Sc-doped SrTiO_3_–Sm_2_O_3_ target was then pressed and sintered just as for the undoped SrTiO_3_ composite target above.

X-ray diffraction characterisation of the films was performed using a four-circle diffractometer and the data was fitted and analysed using X'pert HighScore Plus software suite. An atomic force microscope in tapping mode was used for the AFM studies. The microstructures were then characterized by (scanning) transmission electron microscopy (STEM) (FEI Tecnai G2 F20 operated at 200 kV) to collect the STEM figures used in this study. The polarisation hysteresis loops were acquired by employing Radiant Precision Premier which used a Sawyer-Tower circuit and an in-house made probe station. The impedance analyser Agilent 4294A was used to perform the capacitance, tangent loss *vs.* electric field and capacitance, tangent loss *vs.* frequency studies. An in-house-made heater coupled with the probe station was used to perform the high temperature measurements. An in-house written LabVIEW program was used to collect the data. The *K*-factor was usually computed from the dielectric constant and loss values for 0 kV cm^–1^ and negative maximum values for electric field (*i.e.*, –400 kV cm^–1^, –500 kV cm^–1^, –1000 kV cm^–1^, *etc*.), as the loss was usually the lowest for negative electric fields.

To enable dielectric measurements, Pt sputter-coated top electrode pads of diameter ∼100 μm were deposited on films grown on SrRuO_3_/SrTiO_3_ or Nb-doped SrTiO_3_. A parallel-plate configuration was used for these measurements.

## Conflicts of interest

There are no conflicts to declare.

## Supplementary Material

Supplementary information
