# Supplementary material for: Very high commutation quality factor and dielectric tunability in nanocomposite SrTiO3 thin films with Tc enhanced to >300 °C
Source: Nanoscale. 2018 Feb 5;10(7):3460–8. doi: 10.1039/c7nr06991j (PMC5815283; doi:10.1039/c7nr06991j)
Supplement: Supplementary file 1 [file NR-010-C7NR06991J-s001.pdf]

Electronic Supplementary Material (ESI) for Nanoscale.
This journal is © The Royal Society of Chemistry 2017

Supplementary information

Very High Commutation Quality Factor and Dielectric Tunability in Nanocomposite $SrTiO_3$ Thin films with T_c Enhanced to >300 °C

Abhijeet L. Sangle[1,*], Oon Jew Lee[2], Ahmed Kursumovic[1], Wenrui Zhang[3], Aiping Chen[4], Haiyan Wang[5] and Judith L. MacManus-Driscoll[1,*]

[1]Department of Materials Science and Metallurgy, University of Cambridge, United Kingdom

[2]School of Fundamental Science, Universiti Malaysia Terengganu, 21300 Kuala Terengganu, Malaysia.

[3]Center for Functional Nanomaterials, Brookhaven National Laboratory, Bldg. 735 – P.O. Box 5000, Upton, NY, 11973-5000, United States

[4]Center for Integrated Nanotechnologies (CINT), Los Alamos National Laboratory, Los Alamos, NM 87545, United States

[5]School of Materials Engineering, Purdue University, West Lafayette, IN, 47907, United States

[*]Corresponding authors: as2174@cam.ac.uk, jld35@cam.ac.uk

The highly vertically strained, heteroepitaxial growth of $SrTiO_3$ in $SrTiO_3$-Sm_2O_3 nanocomposite films on *(001)* $SrTiO_3$ substrates is clearly seen from the X-ray diffractogram of Fig. S1(a), where only *(00l)*-type peaks were seen. The Sm_2O_3 phase also grew heteroepitaxially, with 45° in-plane rotation compared to the $SrTiO_3$ substrate, as seen from the X-ray phi-scans shown in Fig. S1(b).

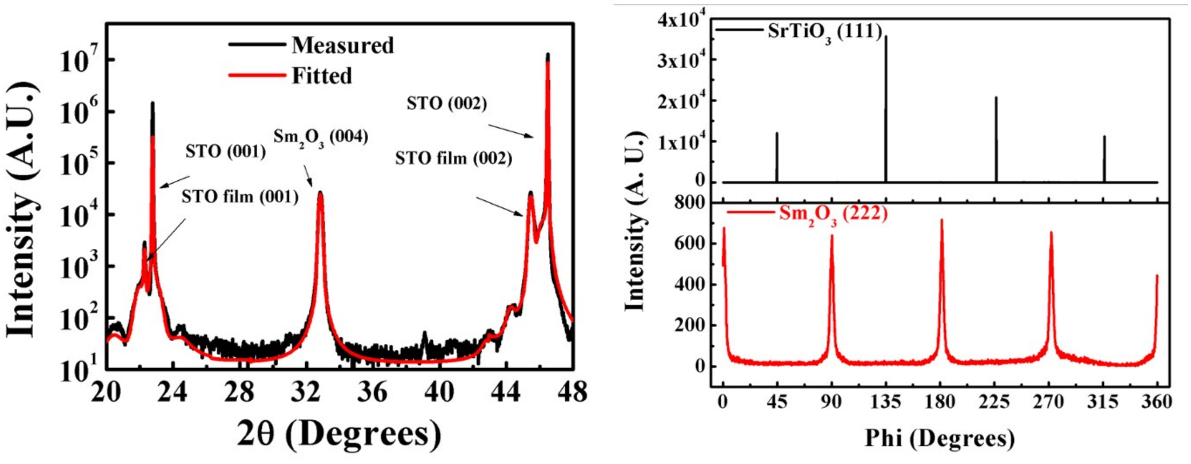

(a) **(b)**

Fig. S1 (a) Out-of-plane symmetric 2θ-ω X-ray diffraction scan of $SrTiO_3$-Sm_2O_3 film, (b) phi scan around $SrTiO_3$ (111) and Sm_2O_3 (222) peaks showing the in-plane epitaxial relationship between the $SrTiO_3$ substrate and the Sm_2O_3 filmTo determine the Curie temperature, polarisation vs. electric (PE) loops were measured as the sample was heated. These results are presented in Fig. S2(a). Fig. S2(b) shows the capacitance (and dielectric loss (tan δ) vs. d.c. electric field measurements performed at room temperature and 1 MHz, up to electric fields of 1000 kV/cm in either direction.

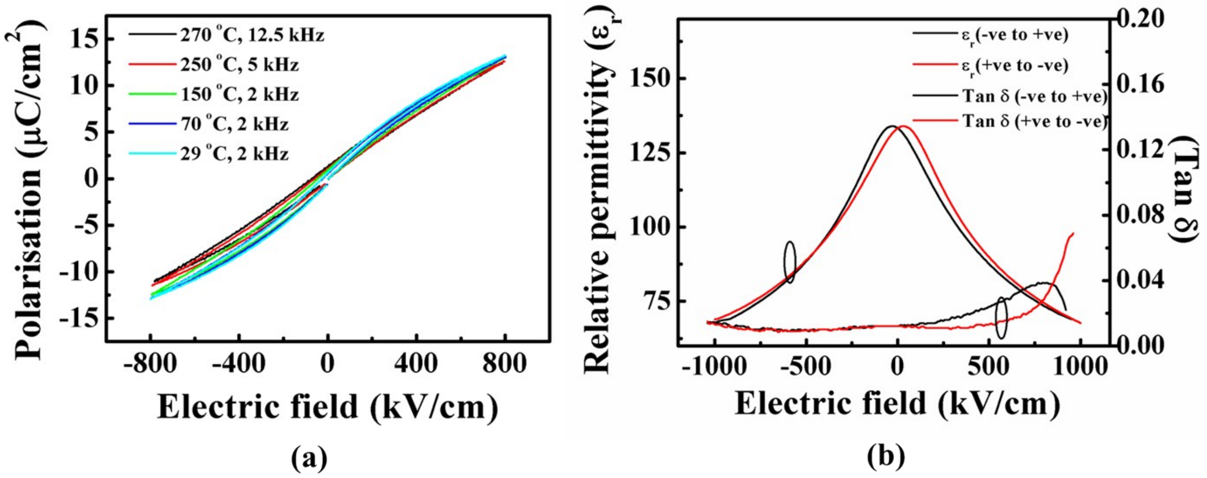

(a) **(b)**

Fig. S2 (a) Polarisation vs. electric field (PE) loops from room temperature to 270°C, and (b) Relative permittivity (ε_r) and Tan δ vs. electric field measurements for a 250 nm thick

$SrTiO_3$-Sm_2O_3 (60 wt.% Sm_2O_3) nanocomposite film grown on Nb-doped $SrTiO_3$ (001) single crystal substrate.

From Fig. S2(a), we can clearly see that the film has retained ferroelectric character at least to 270 °C. Beyond this temperature the loops became quite lossy to conclude their ferroelectric or paraelectric nature. The tunability as calculated from the capacitance vs. electric field measurements shown in Fig. S2(b) was found to be ~49% with a K-factor of 2800.

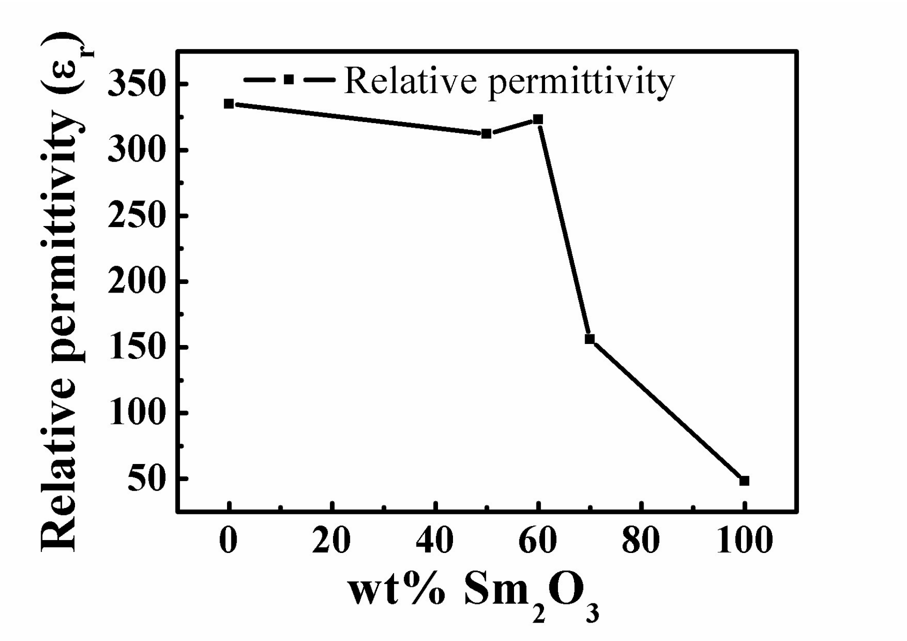

Fig. S3 Variation of the relative permittivity of the columnar composite films at zero DC bias as the Sm_2O_3 content is varied.

From Fig. S3 we see that the relative permittivity (ε_r) of the films decreases with increasing Sm_2O_3 content. This is explained by the lower relative permittivity of Sm_2O_3.
